# Transparency and translation of science in a modern world

**DOI:** 10.1186/1476-069X-12-70

**Published:** 2013-08-27

**Authors:** Philippe Grandjean, David Ozonoff

**Affiliations:** 1 Department of Environmental Medicine, University of Southern Denmark, 5000 Odense, Denmark; 2 Department of Environmental Health, Harvard School of Public Health, Boston, MA USA; 3 Department of Environmental Health, Boston University School of Public Health, Boston, MA USA

**Keywords:** Decision Making, Environmental Health Science, Open Access Publishing

## Abstract

The co-Editors-in-Chief of *Environmental Health* respond to an unusual initiative taken by editors of 14 toxicology journals to influence pending decisions by the European Commission to establish a framework for regulating chemicals that pose a hazard to normal function of the endocrine system. This initiative is also the subject of this Commentary in this journal by authors who recently reviewed the subject and who point out inaccuracies in the toxicology editors’ critique. The dispute is about potential public policy development, rather than on science translation and research opportunities and priorities. The toxicology journal editors recommend that chemicals be examined in depth one by one, ignoring modern achievements in biomedical research that would allow new understanding of the effects of classes of toxic substances in complex biological systems. Concerns about policy positions framed as scientific ones are especially important in a time with shrinking public support for biomedical research affects priorities. In such a setting, conflict of interest declarations are important, especially in research publications that address issues of public concern and where financial and other interests may play a role. Science relies on trust, and reasonable disclosure of financial or other potential conflicts is therefore essential. This need has been emphasized by recent discoveries of hidden financial conflicts in publications in toxicology journals, thus misleading readers and the public about the safety of particular industrial products. The transparency provided by *Environmental Health* includes open access and open peer review, with reader access to reviews, including the identity of reviewers and their statements on possible conflicts of interest. However, the editors of the 14 toxicology journals did not provide any information on potential conflicts of interest, an oversight that needs to be corrected.

## Science informing policy

In a Commentary published in this journal, Bergman et al. [1] respond to a highly unusual coordinated set of identical editorials in 14 toxicology journals, now available ahead-of-print [2]. The parallel editorials in these scientific journals are not about specific research findings, nor existing science-based public policy. Instead they are written with the sole purpose of influencing pending policy decisions of the European Commission. At stake is the future regulatory framework for industrial chemicals suspected of affecting functions of the human endocrine system, a key player in development and physiological function and also a key to the pathogenesis of important non-communicable diseases [3,4].

The essence of the position of the toxicology journal editors is that there is insufficient evidence to justify any new regulation regarding effects of chemicals on the endocrine system. They further endorse the general strategy that risk assessments of the tens of thousands of untested chemicals be conducted separately for each, one at a time. This conclusion reminds us of the unfortunate advice another group of toxicology experts gave more than 20 years ago in regard to developmental toxicology: “Differences in sensitivity between children and adults are chemical specific and must be studied and evaluated on a case-by-case basis” [5]. The reluctance to accept that children and the fetus are often much more vulnerable to toxicants than are adults, regulation of industrial chemicals, such as lead and mercury, was delayed by many years, if not decades, thereby causing harm to untold numbers of children [6]. We are concerned that such advocacy of particular solutions belongs within the policy-development realm, not within toxicology or the science-based translation of toxicology, notwithstanding the fact that the editorial is written by editors of science journals.

Dietrich et al. assert (without supporting citation) that the proposed legal framework deliberately ignores or is ignorant of time-tested principles of the science of toxicology that have been universally accepted for centuries [2]. They offer as their model the early 20^th^ century whole organism assay (either human or laboratory animal) that was the mainstay of a much older generation of toxicologists. This view was prevalent before the discovery and deciphering of the genetic code and before the first hormone protien was sequenced (1953) or radioimmunoassay (1960) ushered in a new era in endocrinology. At about the same time, compartment analysis and mathematical modeling of systems with feedback loops became possible, but the more complicated biological systems remained relatively intractable until methods for qualitative analysis of systems of coupled non-linear differential equations and chaotic systems became prevalent starting in the 1980s. Recently these analytical methods have been linked with modern genomics, epigenetics, and microanalysis of biological compounds, thereby revealing a New World of effects and consequences unforeseen by classical toxicology.

Modern endocrinology has therefore seen a paradigm change prompted by modern methods of science. However, the view of the editors of the journals represented by Dietrich et al. [2] appears to be stuck in the last century, before recent scientific achievements. The authors seem to have missed the great advantages of computational chemistry, gene expression and receptor binding assays, knock-out animal models, and many other accomplishments that now inform modern endocrinology. Dietrich et al. still promote a focus on individual substances to generate solid understanding of each of their properties in isolation only on whole organisms, rather than utilizing our new understanding of the effects of toxic substances in complex biological systems.

Thus, the methods and teachings that underlie the editorial by Dietrich et al. [2], while not cited, are implicitly the methods and teaching of a previous generation of toxicologists. They also bear no relation to modern endocrinology, which may not be published in toxicology journals but in other specialty journals. As the Commentary by Bergman et al. [1] points out, to make matters worse, in our view the journal editors also misstate the scientific positions of the European Commission, WHO and numerous other international bodies that have considered this matter.

But the editorial by Dietrich et al. [2] is not really about science, whether contemporary or old fashioned. It is explicitly about public policy. It can conflate the two only by claiming the science as settled, a product of centuries of accepted methods and established teaching, although it gives no evidence to substantiate this sweeping and inaccurate claim. While the science that comprises the context of the pending Commission decisions is modern, the public policy question is not: it is the problem of taking important decisions in the face of varying levels of uncertainty [7]. Unlike Dietrich et al., we acknowledge that this uncertainty exists. It is the responsibility of policy makers, not scientists, to shape the policy in a way that the net benefit is positive and maximized, taking account of societal values and norms. Such decisions have potential economic and societal consequences. Often industry and society adapt and the disadvantages are minor, perhaps even promoting innovation to the benefit of industry, as suggested in a recent commentary on climate change [8]. Dietrich et al. [2] assume that the consequences are “profound,” but give no evidence to support their black-and-white view.

Apart from these differences in perspectives, there are some important links to science policy that should not be left uncommented upon. The demand that chemicals be considered one at a time comes in a context where public funding for research is contracting. In the EU, the plans for Horizon 2020 suggest that the funding for biomedical research will fall while it becomes even more focused, and the US biomedical research budget is shrinking for the first time in its history. Private corporations are at the same time voicing concerns about the burden of having to test their chemical products. In this context, the arduous and time-consuming task of chemical-by-chemical evaluation by classical standards of toxicology is not just a delay in providing vital information about the safety of our environment but, practically speaking, a denial of such testing as a matter of policy. The view of Dietrich et al. [2] is therefore, not a prescription for better information but a prescription for dramatically less information relative to the enormous task at hand.

## Conflicts of interest

Whatever the course of action, there will be trade-offs. Policy decisions are not just about a simple balancing of risks and benefits. Usually the risks fall disproportionately on those who do not accrue the most benefits. In such a setting, conflicting goals are to be expected and it is in just such a setting that transparency about conflicts of interest becomes paramount. Editors of science journals should be beyond such conflicts, and their decisions on their colleagues’ manuscripts should be neutral and impartial. We realize that this is not necessarily always the case [9], but any deviation from the ideal should at least be transparent and obvious to the reader. Major scientific journals follow international recommendations on conflict of interest declarations in regard to authors, reviewers, and editors [10]. At least this is what the journal websites say. But precisely because conflicts mean that there could be an interest in keeping them secret, we don’t know how often conflicts of interest are hidden in violation of the formal journal policies.

In July of this year, a court case revealed that the four independent laboratories who contributed to a peer-reviewed article in one of the toxicology journals may not have been as independent as the publication suggested [11]. The paper aimed at documenting the lack of endocrine disrupting properties of a plastic material. The authors declared no conflict of interest and there was no Acknowledgement section in the article. When the scientific methods and interpretation were challenged by a competing company it was revealed that the producer of the chemical in question had designed the study, paid the first author to generate the manuscript and covered all expenses by the participating laboratories. This information was withheld from the reader. In our view, this is a serious breach of trust between authors, editors, and readers. Readers of science publications and books should know who initiated the science, who paid for it, and who wrote the manuscript. It may be that this information is of no consequence, but accepted practice dictates that it be disclosed, while hiding it suggests that there could be something unsavory going on.

Unfortunately, since the information is withheld it is especially difficult to find out how often this occurs. Some insight into hidden conflicts of interest stems from documents obtained at trials, particularly those involving tobacco companies or drug companies [12]. In some recent articles on asbestos, authors erroneously indicated that their research was supported by a “grant” from a particular company, suggesting that the researchers had independence to explore the research questions [13]. In reality, several publications were funded by hourly honoraria for consulting services, and manuscripts were drafted by company employees before co-authors were approached. An appellate court opinion in June of 2013 referred to this practice as potentially being criminally fraudulent [14]. A total of eleven articles with misleading statements on conflicts of interest appeared in four different journals that scientists in the field would consider reputable sources. One toxicology journal that published four of the articles released “Corrigenda” [15] and clarified that one author was employed by a company with direct interest in the results and that “other authors are consulting experts retained by or on behalf of [the company] to conduct the research and prepare the articles.” This carefully worded text still leaves the readers in the dark about who did what and how the company interests affected the research. Perhaps the reviewers and editors were also misled. None of the articles has been retracted.

The portfolio of the publisher of the journal in question also includes another toxicology journal where one of us (PG) served for many years on the editorial board. Last year, the journal published several articles timed to coincide with regulatory initiatives regarding endocrine disruptors and other human health hazards with a clear bias toward industry interests, thus provoking a discussion on conflicts of interest. The publisher offered to conduct a thorough review of all manuscript authors and reviewers to determine whether a bias was present. However, the publisher failed to act on the promise, resulting in an end to a long-term collaboration.

In general terms, a conflict of interest exists when an author “has financial or personal relationships that inappropriately influence (bias) his or her actions”. Although remedies exist to close a widespread credibility gap with industry-sponsored research, they are meaningful only in connection with transparency. The problem of hidden agendas has magnified as the power of science to legitimate regulation of pollutants has become more obvious and academic research increasingly dependent on industry support [16]. Thus, science has now become part of a war, although most of the battles are fought behind the scenes.

We emphasized in our recent Editorial [17] that trust is essential in research. While we have accepted practices in place to minimize undisclosed conflicts, we remain vigilant because the recent events regarding concealed conflicts of interest suggest that we may not receive the full information about potential conflicts. We are aware of only a single case where a paper published in *Environmental Health* had a potential undeclared conflict. In that instance, an immediate correction was published. We believe that open peer review and open access provide additional safeguard, as readers have access to the peer reviews and information on possible conflicts of interest among both authors and peer reviewers. But it is no guarantee and we will continue to be on the alert.

Trust is also a necessary condition, upon which the links between scientists, journal editors, reviewers, publishers, and, ultimately, the public rely. Perhaps it was an oversight, or perhaps Dietrich et al. believe that editors do not need to provide statements on conflicts of interest. Their editorial [2] did not include any statement on Competing Interests that might have elucidated whether they have personal conflicts that would be affected by potential European Commission decisions they have taken so much time and effort to oppose even before they have been enacted. We urge Dietrich et al. to correct that lapse.

## Competing interests

PG and DO are founding editors-in-chief of *Environmental Health* but have no other interests to declare.

## Authors’ contributions

PG drafted the first version of the manuscript, and DO and PG both contributed to and approved the final version.
